# Influence of Structural Theories on Optimal Fiber Distributions in Tow-Steered Composites Considering Local Strain and Stress

**DOI:** 10.1007/s42496-025-00275-3

**Published:** 2025-05-26

**Authors:** A. Pagani, A. Racionero Sánchez-Majano, M. Petrolo

**Affiliations:** MUL2 Lab, Department of Mechanical and Aerospace Engineering, Politecnico di Torino, Corso Duca degli Abruzzi 24, 10129 Turin, Italy

**Keywords:** Variable stiffness composites, Variable-angle tow, Unified formulation, Structural optimization

## Abstract

Tow-steered composites offer the possibility to tailor and enhance the mechanical performance of lightweight structures thanks to their larger design space compared to straight-fiber composites. This work proposes a scalable low- to high-fidelity methodology to retrieve the fiber orientations that optimize strain and stress distributions in variable stiffness plates. An optimization algorithm that combines global and local search strategies solves unconstrained and manufacturing-constrained problems. The structural models are generated through the Carrera Unified Formulation, which permits tuning the accuracy of the solution by selecting the order of the structural theory employed. The results show differences in the optimal stacking sequences as free-edge effects, local distortions, and 3D stress states are involved in the objective functions. Additionally, differences in the prediction of the quantities of interest are found between low-to-refined equivalent-single-layer—including the particular cases of the classical plate theory and the first-shear order deformation theory—and high-fidelity layer-wise models.

## Introduction

Continuous improvements in manufacturing technologies have allowed the conception of new families of composite materials. For instance, braided composites that improve impact resistance [1, 2]. Another type of composite that has been gaining the attention of researchers for the past ten years is the so-called Variable Stiffness (VS) or Variable Angle Tow (VAT) composites. Despite this recent interest, the VS concept is not new and was introduced first by Leissa and Martin [3]. They studied the buckling and free vibration response of straight-fiber composites in which the fibers were non-uniformly distributed within the lamina. They found an improvement of up to 38% and 21% in such characteristics, respectively. Later, Gürdal and Olmedo [4] proposed a linearly varying pattern of the fiber angle orientation. Therein, closed-form solutions based on the Classical Laminated Plate Theory (CLPT) were proposed for the in-plane response of VAT plates. Likewise, Gürdal et al. [5] studied the buckling of VAT laminates and conducted a parametric analysis depending on different boundary conditions and rotations of the fiber paths. They showed that, depending on the fiber path rotation angle, the buckling load can be improved up to 19% and 80% compared to classic straight-fiber laminates. These solutions were obtained by numerically solving the set of partial differential equations based on the CLPT. In the middle 1990s, Waldhart et al. [6] compared VAT in which consecutive fiber paths were shifted and parallel. They demonstrated that both manufacturing approaches lead to higher buckling loads than classic straight-fiber laminates. Nonetheless, they concluded that shifted courses redistribute the loading better than parallel fiber paths. CLPT-based partial differential equations were solved numerically to conduct the analysis. Note that these seminal works [4–6] did not consider optimization of VS laminates but rather performed parametric studies of fiber paths and structural theories to understand the behavior of such components.

The mechanical characteristics of composite structures can be tailored to the desired specifications by finding the stacking sequence that maximizes or minimizes a desired structural feature. Several methods have been proposed throughout the years to solve the optimization problem. For instance, Haftka and Walsch [7] proposed an integer programming scheme to maximize the buckling load of straight-fiber laminates. Afterward, Le Riche and Haftka [8] introduced an integer-valued Genetic Algorithm (GA) to maximize the buckling performance. On the one hand, GA eases accounting for the manufacturing constraints commonly imposed in optimizing straight-fiber composite laminates; see [9]. On the other hand, the integer-valued GA scheme poses an intricate non-convex optimization problem. Fukunaga and Sekine [10] introduced the lamination parameters to solve this issue. The expressions of the lamination parameters constrain the design space to determine the feasible convex region where laminate configurations exist. These expressions rely on the CLPT and First-order Shear Deformation Theory (FSDT). Silva et al. [11] combined GA and lamination parameters to conceive the Slice and Swap Method for the aeroelastic tailoring of regional aircraft. Slice and Swap is a two-step optimization problem in which (i) a continuous optimization provides the thickness and directional stiffness distribution over the whole wing that satisfies the safety margins; (ii) a discrete optimization step retrieves the combination of stacking sequences that match the directional stiffness from the previous step, and that satisfies all the design and manufacturing rules. As in [11], Catapano et al. [12] developed a two-level optimization strategy to obtain optimized VAT designs manufactured by Fused Filament Fabrication (FFF) and Continuous Filament Fabrication (CFF). Similarly, two-level approaches utilizing gradient-based optimization algorithms for the strength and mass optimization of variable stiffness composites were proposed by Izzi et al. [13, 14] where the design variables are the thickness and the polar parameters of the laminated structure. In addition, the work by Gandhi et al. [15] proposed a gradient-based approach like the geometry projection method that can be utilized for the topological optimization of additive-manufactured VSCs. They used FSDT as the structural theory as they considered thin plates in their analyses, and used the discrete material optimization approach with the solid isotropic material with penalization for laminated structures subject to initial excitation for minimum residual vibration [16]. As in the previously cited works, they employed FSDT to model the laminates. The above optimization strategies can be applied to retrieve the optimal stacking sequence of VAT laminates. For example, Serhat and Basdogan [17] used CLPT lamination parameters to calculate the optimal fiber paths and imposed manufacturability by calculating the curvature radius point-wise. Conversely to straight-fiber laminates, in which orientations are limited to a fixed number, in VSC composites, the parameters that define the fiber path are continuous. Thus, encoding is unnecessary if employing GA or any other evolutionary algorithm like Particle Swarm Optimization (PSO). Singh and Kapania [18] maximized the buckling load of curvilinearly stiffened VAT plates through a PSO algorithm. The design variables were the fiber path orientation parameters and the parameters employed to define the shape of the stiffeners. They used Nastran shell elements for the laminates, whereas the stiffeners were modeled with Nastran beam elements. Groh and Weaver [19] minimized the weight of VAT plates manufactured with the Continuous Tow Shearing (CTS) process [20]. GA performed the mass optimization, including static failure and buckling constraints. At the same time, the Kirchhoff plate model was considered along with von Kármán non-linear strains for the structural problem. Vijayachandran et al. [21] faced the multi-objective optimization of VSC components. The in-plane stiffness, buckling load, and mass were the objective triplets to be considered. In this work, the fiber paths were generated employing Bézier curves, and the design variables were the control points of the curves. Shell-like S4R Abaqus Finite Elements (FE) were used to model the VAT structure.

A recursively faced problem in composites is the study of stress concentration factors around cutouts. It is paramount in aerospace, since cutouts accommodate windows, doors, and bolted joints. Related to VAT composites, Hyer et al. [22, 23] investigated the improvement of buckling resistance and tensile load in the presence of a hole. Senocak and Waas [24] proposed to nullify the stress concentration by introducing a reinforcement around the hole that maintained the same moment and curvature distributions as of the uncut plate. Zappino et al. [25] conducted an experimental and virtual test campaign to assess the stress concentration factor on 3D-printed coupons of both isotropic and orthotropic materials. Strain fields predicted with FE showed to be in excellent agreement with those obtained during the experimental test, which was acquired using a digital image correlation technique. Lopes et al. [26] tailored the buckling and post-buckling first ply failure of a cutout panel, showing advantages compared with constant-stiffness laminates.

The stiffness-based tailoring, depicted in the previous paragraph, has usually been used as a substitute for strength-based optimization. The objective of the strength-based optimization is to minimize the failure index, which is equivalent to maximizing the safety factor, as stated by Groenwold and Haftka [27]. They compared the stiffness- and strength-based optimization for different materials and loading conditions. The results showed a favorable correlation between stiffness- and strength-driven designs, but it depends on the material properties and the loading conditions. The CLPT was utilized as the structural model, and Tsai-Wu and Tsai-Hill [28] failure criteria were considered. Ijsselmuiden et al. [29] implemented the Tsai–Wu failure criterion in the lamination parameter design space. They derived a conservative failure envelope that guaranteed a failure-free region of the lamination parameter space regardless of the fiber orientations involved in the laminate’s stacking sequence. The methodology described initially in [29] was extended to VAT composites in the work by Khani et al. [30]. They considered the lamination parameter for each node of the FE model, increasing the number of design variables compared to a straight-fiber laminate. The results showed a threefold and twofold improvement compared to a quasi-isotropic laminate and a stiffness-driven VAT design. As in [27, 29], Khani et al. [30] considered CLPT. In these works, the lamination parameters were considered as the design variables. However, the continuity of the fiber paths is not guaranteed a priori, and reconstruction is needed.

The literature review demonstrates that equivalent-single-layer (ESL) classical theories have been typically used to optimize VAT components, and very few works considered a layer-wise (LW) approach for structural modeling. LW theories are developed by postulating that the displacement field exhibits $C^0_z$-continuity through the laminate thickness [31]. This work considers both approaches for the optimization process. The Carrera Unified Formulation (CUF) [32] is employed to generate ESL and LW models. In recent years, CUF has been used to create both ESL and LW models to analyze VAT plates. For instance, Demasi et al. [33] employed 2D ESL, Zig-Zag, and LW models for the stress analysis of thick VSC laminates. Moreover, shell structures with spatially varying fibers were investigated in [34]. The through-the-thickness stress distribution using both ESL and LW approaches was predicted. As expected, LW outperformed ESL when predicting transverse stresses. Finally, buckling and fundamental frequency optimization were faced in [35]. Results showed that, for thin laminates, ESL and LW lead to similar values in both the design variables and quantities of interest. In contrast, the optimal design variables were similar for thick plates, but discrepancies were present in the prediction of the objective function. Besides its application to tow-steered composites, CUF has successfully proven its versatility in computational mechanics. For instance, free-edge analysis of laminated structures [36], micromechanics [37], and non-local theories for damage modeling [38].

This work proposes the optimization of defect-free VAT plates modeled with CUF-based LW models. It investigates the effect of the chosen structural theory on the retrieved optimal design variables for strain and stress optimizations. These problems are solved by an algorithm that combines global and local search strategies, and manufacturing constraints, i.e., the fiber path curvature, are considered. The paper is organized as follows: Sect. 2 presents the variable stiffness concept and manufacturing constraints; the unified formulation and the finite elements employed to model the laminated structures are introduced in Sect. 3; Sect. 4 provides the model verification and the results for the optimization problems. Conclusions are drawn in Sect. 5.

## Variable Stiffness Laminates and Manufacturing Constraints

**Fig. 1:**
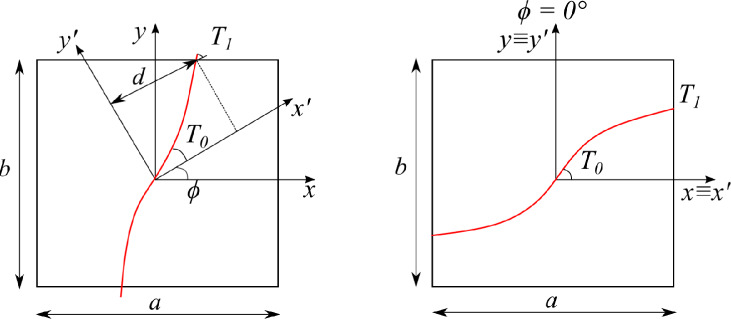
Graphical representation of the parameters involved in a linearly varying fiber path for $\phi \ne 0^\circ$ (left) and $\phi =0^\circ$ (right)

VAT laminates are fabricated by steering fiber bands, called courses, along curvilinear paths. Over the years, several variation laws have been investigated, the most adopted ones with a constant curvature and those with a linear variation. This manuscript focuses on the latter, in which the local fiber orientation of the $k^{th}$ layer, $\theta ^k$, varies along the $x'$ direction as follows:

1 $$\begin{aligned} \theta ^k(x')=\phi ^k+T_0^k+\frac{(T_1^k-T_0^k)}{d}|x'|. \end{aligned}$$

Therein, $T_0^k$ is the fiber angle orientation at $x'=0$, $T_1^k$ denotes the fiber angle when $x'=d$, in which *d* is the length along which the fiber orientation varies from $T_0^k$ to $T_1^k$; last, $\phi ^k$ is the fiber path rotation angle and defines the axis along which the courses are steered, i.e., *x*-axis, *y*-axis, or a combination of both. Indeed, the $x'$ axis is expressed as $x'=x\cos \phi +y\sin \phi$. These parameters are illustrated in Fig. 1. Note that *d* typically corresponds to the semi-length or semi-width of the plate, as the $\phi ^k$ rotation angle often is $0^\circ$ or $90^\circ$, respectively. However, the authors suggest using $d=\sqrt{(a/2)^2\cos ^2\phi ^k+(b/2)^2\sin ^2\phi ^k}$ for the sake of generality.

The fabrication of tow-steered laminates presents limitations, one of them being the curvature of the laid fiber path. Otherwise, fiber wrinkles or upfolding will be generated within the placed tape. Hence, there is a limitation in the fiber paths that can be obtained for each lamina. In the optimization problem considered in this paper, the curvature is a constraint. A commonly adopted minimum radius for carbon-fiber reinforced plastics is $r_{\text {min}}=0.635$ m [39–41]. Nonetheless, depending on the tape width and the manufacturing process, a lower radius of curvature may be achievable. Thus, the curvature of the $k^{th}$ ply is constrained as

2 $$\begin{aligned} -\frac{1}{r_{\text {min}}}\le \kappa ^k \le \frac{1}{r_{\text {min}}}. \end{aligned}$$

**Fig. 2:**
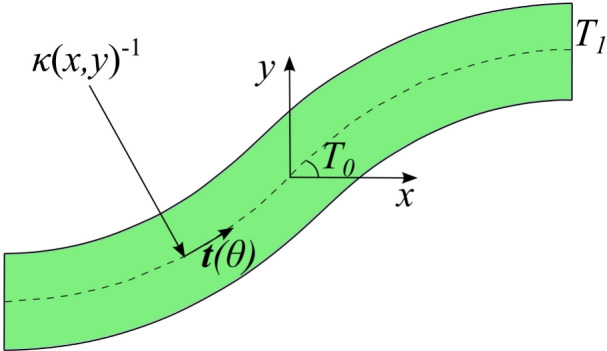
Graphical representation of the AFP turning radius and unitary tangent vector in a linearly varying fiber path at (*x*, *y*) in-plane coordinates

The curvature of a generic fiber path can be derived following the methodology presented in the work by Brooks and Martins [42] by defining the unit tangent vector ${\textbf{t}}$, see Fig. 2., of the fiber path as

3 $$\begin{aligned} {\textbf{t}}(\theta )=\cos (\theta ){\hat{\textbf{i}}} + \sin (\theta ){\hat{\textbf{j}}}. \end{aligned}$$

Then, computing the curl operator over the vector field ${\textbf{t}}$ and keeping the only nonzero vector component yield

4 $$\begin{aligned} \kappa (x,y)=(\nabla \times {\textbf{t}}(\theta ))\cdot {\hat{\textbf{k}}}= \frac{\partial \theta }{\partial x}\cos (\theta )+\frac{\partial \theta }{\partial y}\sin (\theta ), \end{aligned}$$

which can be evaluated to asses the manufacturing feasibility of the design. The symbols ${\hat{\textbf{i}}}$, ${\hat{\textbf{j}}}$, and ${\hat{\textbf{k}}}$ denote the unitary vectors of a Cartesian reference frame. Note that in the case of a fiber varying along the *x*-direction, i.e., $\phi =0^\circ$, Eq. (4) reads as follows:

5 $$\begin{aligned} \kappa ^k(x)=\text {sgn}(x)\frac{(T_1^k-T_0^k)}{d}\cos \left( T_0^k+\frac{(T_1^k-T_0^k)}{d}|x|\right) , \end{aligned}$$

where sgn$(\cdot )$ denotes the sign function. The curvature of a fiber path is illustrated in Fig. 2. Modern AFP and additive manufacturing technologies enable more advanced patterns. Linearly varying fiber paths were considered, because the paper aims to investigate the influence of the structural theory on the optimization solution, i.e., how the use of low-fidelity theories may hinder the optimization process by providing wrong stress values. Such wrong estimations are expected to impact the optimization independently of the fiber path adopted. In future research, more complex patterns, such as those based on Bézier curves or Non-Uniform Rational B-Splines, could be explored, as in [13, 14, 21]. Other limitations in the fabrication of tow-steered composites are the occurrence of defects such as gaps and overlaps. These are commonly referred to as the manufacturing signature [21] of the steering process, and their study is of utmost importance when analyzing VAT laminates. Some studies include these defects in the numerical model used during the optimization process [21], while others try to limit their presence by imposing additional constraints [42]. These defects are not considered in the present document, as it is not the scope of this research.

## Higher-Order 2D Plate Theories

The CUF is used to implement 2D FE. According to [32], the 3D field of displacements can be expressed in terms of arbitrary through-the-thickness expansion functions, $F_\tau (z)$, of the 2D generalized unknowns laying over the $x-y$-plane. That is

6 $$\begin{aligned} {\textbf{u}}(x,y,z) = F_{\tau }(z){\textbf{u}}_{\tau }(x,y) \hspace{5.0pt}\tau =1,..,M, \end{aligned}$$

being *M* the number of expansion terms and ${\textbf{u}}_{\tau }(x,y)$ is the vector containing the generalized displacements. Note that $\tau$ denotes summation. In particular, this document employs Taylor and Lagrange polynomials, referred to as Taylor expansion (TE) and Lagrange expansion (LE), respectively. An example of a third-order TE is reported in the following:

7 $$\begin{aligned} \begin{aligned}&u_x = u_{x1}+zu_{x2}+z^2u_{x3}+z^3u_{x4},\\&u_y = u_{y1}+zu_{y2}+z^2u_{y3}+z^3u_{y4},\\&u_z = u_{z1}+zu_{z2}+z^2u_{z3}+z^3u_{z4}. \end{aligned} \end{aligned}$$

In contrast, a second-order LE reads as follows:

8 $$\begin{aligned} {\textbf{u}}=L_1(r){\textbf{u}}_{1}+L_2(r){\textbf{u}}_{2}+L_3(r){\textbf{u}}_{3}, \end{aligned}$$

where

9 $$\begin{aligned} L_1(r)=\frac{1}{2}\left( r^2-r\right) , \hspace{5.0pt}L_2(r)=\left( 1-r^2\right) , \hspace{5.0pt}L_3(r)=\frac{1}{2}\left( r^2+r\right) , \end{aligned}$$

in which $L_i$ are the Lagrange polynomials defined in the natural reference frame exploiting the isoparametric formulation. Following this notation for the expansion function, TE*n* indicates a TE of $n^{th}$-order, while LE*n* represents the use of LE with $n^{th}$-order polynomials. Furthermore, *X*LE*n* denotes the use of *X* $n^{th}$-order Lagrange polynomials to describe each laminate layer.

Utilizing the FE and shape functions $N_i(x,y)$, the displacement field becomes

10 $$\begin{aligned} {\textbf{u}}_\tau (x,y)=N_i(x,y)F_{\tau }(z){\textbf{q}}_{\tau i}\hspace{5.0pt}\hspace{5.0pt}i = 1,\ldots ,N_n, \end{aligned}$$

where ${\textbf{q}}_{\tau i}$ denotes the unknown nodal variables, and $N_n$ indicates the number of nodes per element. 2D nine-node quadratic, Q9, elements are employed as $N_i$ for the $x-y$-plane discretization.

The principle of virtual displacements (PVD) is used to derive the governing equations of the FE model. The PVD states that in a static problem, the virtual variation of the internal strain energy, $\delta {\mathcal {L}}_{int}$ , has to be equal to the virtual work of the external forces, $\delta {\mathcal {L}}_{ext}$, that is

11 $$\begin{aligned} \delta {\mathcal {L}}_{int}=\delta {\mathcal {L}}_{ext}. \end{aligned}$$

The virtual variation of the strain energy can be calculated as

12 $$\begin{aligned} \delta {\mathcal {L}}_{int}=\int _V\delta \boldsymbol{\varepsilon }^{k^T}\boldsymbol{\sigma }^kdV. \end{aligned}$$

Equation (12) can be rewritten using Eq. (10), the constitutive law $\boldsymbol{\sigma }^k={\textbf{C}}^k\boldsymbol{\varepsilon }^k$, and the geometrical relations between strains and displacements, thus yielding

13 $$\begin{aligned} & \delta {\mathcal {L}}_{int}=\delta {\textbf{q}}_{sj}^T\left[ \int _V{\textbf{D}}^T(N_jF_s){\bar{\textbf{C}}}{\textbf{D}}(N_iF_\tau )dV\right] {\textbf{q}}_{\tau i} \nonumber \\ & \quad =\delta {\textbf{q}}_{sj}^T{\textbf{K}}^{kij\tau s}{\textbf{q}}_{\tau i}, \end{aligned}$$

where ${\textbf{K}}^{kij\tau s}$ is the 3$\times$3 Fundamental Nucleus (FN) of the stiffness matrix associated with the $k^{th}$ layer of the laminate, which is invariant of the order of the 2D shape functions and the through-the-thickness expansion, as shown in [32]. ${\textbf{D}}(\cdot )$ is the differential operator matrix containing the geometrical relations, and ${\bar{\textbf{C}}}$ is the material stiffness matrix in the global reference frame, i.e., ${\bar{\textbf{C}}}={\textbf{T}}(x,y)^{k}{\textbf{C}}{\textbf{T}}(x,y)^{k^T}$. Note the dependency of the rotation matrix ${\textbf{T}}$ on the in-plane coordinates due to the VAT fiber paths; see [43].

The virtual variation of the external forces is calculated as

14 $$\begin{aligned} \delta {\mathcal {L}}_{ext}=\delta {\textbf{q}}_{sj}^T\int _V {\textbf{p}} N_j(x_p,y_p)F_s(z_p)dV, \end{aligned}$$

in which ${\textbf{p}}$ is a 3$\times$1 array containing the external forces, and $(x_p,y_p,z_p)$ are the coordinates of the point where the force is applied.

**Fig. 3:**
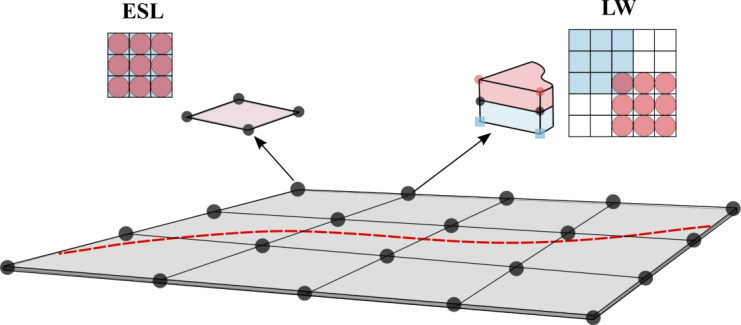
Assembly procedures for ESL and LW models

Looping on the thickness expansion indices, $\tau$ and *s*, and the FE indices, *i* and *j,* of ${\textbf{K}}^{kij\tau s}$, one can obtain the structure’s stiffness matrix ${\textbf{K}}$. The assembly of ${\textbf{K}}$ varies depending on the chosen modeling approach. The Equivalent-Single-Layer (ESL) and Layer-Wise (LW) models are common strategies for analyzing multi-layered structures. In ESL, the homogenization of the properties of each layer is carried out and summed altogether when computing the stiffness matrix. As addressed by Carrera [44], ESL models do not fulfill the $C_{z}^{0}$ requirements. Conversely, LW considers each layer independently and expands the displacement field within each lamina. Consequently, the continuity of displacements has to be imposed at the interface, see [45, 46], thus guaranteeing the completion of the $C_{z}^{0}$ requirements. These two assembly approaches are displayed in Fig. 3 for the case of a plate with two layers. This document employs Taylor polynomials as $F_\tau$ along the thickness direction to generate ESL models. On the other hand, LW uses Lagrange polynomials over the single layers and then imposes the displacement continuity at the interfaces; see [46].

## Numerical Results

### Model Verification

**Fig. 4:**
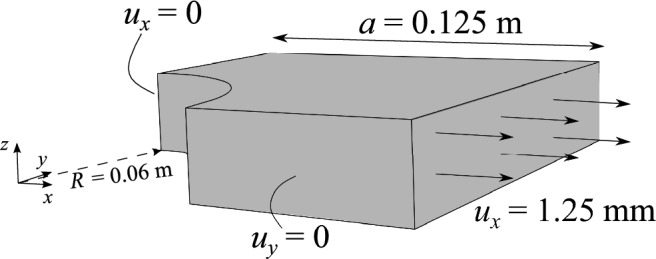
Geometry and boundary conditions of the notched plate

The mathematical framework is verified against the results provided by the commercial software Abaqus (ABQ). The structure under investigation consists of a perforated VAT plate, with semi-width $a=0.125$ m and width-to-thickness ratio $2a/h=10$, corresponding to a relatively thick plate. Table 1 shows the material’s elastic properties. A uniaxial displacement is applied at $x=a$ as illustrated in Fig. 4. Due to the symmetry of loading and geometry, only a quarter of the plate is analyzed by imposing symmetry boundary conditions at planes $x=0$ and $y=0$. Because of the symmetry, the fiber paths need to be mirrored with respect to the $x-z$ and $y-z$ planes.

**Table 1:** Material properties of the perforated VAT plate [21]

$E_{1}$ [GPa]	148.24
$E_{2}=E_{3}$ [GPa]	8.48
$G_{12}=G_{13}$ [GPa]	3.94
$\nu _{12}=\nu _{13}$ [-]	0.329

The concentration of stresses and strains around a plate’s cutout has been widely investigated, and closed-form solutions of the stress concentration factor have been derived for both isotropic and composite materials [47, 48]. However, this factor depends on many geometrical and physical parameters and specific hypotheses regarding the constitutive model and kinematic assumptions. This makes no analytical solutions available in the case of tow-steered composite plates. Thus, this document verifies the numerical model by comparing the $\varepsilon _{xx}$ strain distribution along the *y*-direction calculated with the present methodology and ABQ.

**Fig. 5:**
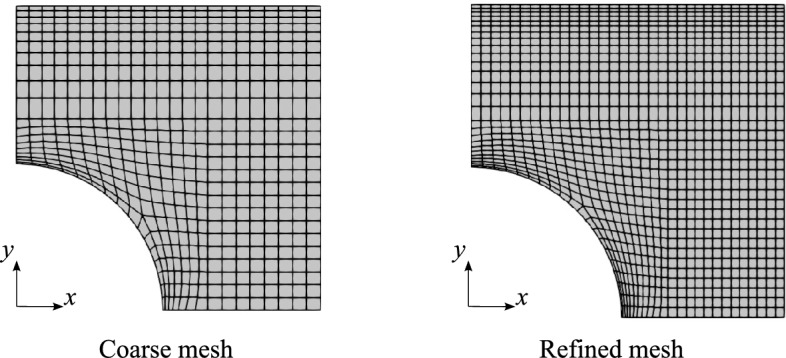
FE meshes used for the convergence study. These meshes are used for the present methodology and the ABQ2D models

**Fig. 6:**
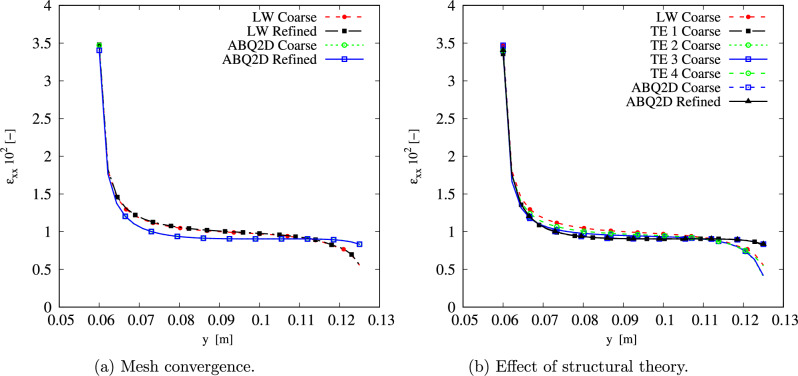
$\varepsilon _{xx}$ distribution for different meshes (a) and effect of the structural theory (b). The LW meshes employ 1LE3 per layer

To verify the model, the $[0\pm \langle 0,15\rangle ]_s$ stacking sequence is assigned to the described geometry, and the results are compared to those obtained with ABQ, as depicted in Fig. 5. The ABQ models use quadratic S8R 2D elements and two different refinement levels. The variation along the *y*-axis of $\varepsilon _{xx}$ for the different ABQ and CUF LW models is displayed in Fig. 6a. Besides, the peak value is listed in Table 2. A good agreement is found for the $\varepsilon _{xx}$ distribution and peak value, recalling that the ABQ model employs an FSDT theory of structures. In contrast, an LW kinematic model is used in the current approach. Moreover, as shown in Table 2, the LW Coarse provides practically the same $\varepsilon _{xx}$ as LW Refined, with a twofold reduction in terms of DOF. Hence, the LW Coarse model is used to study the effect of the through-the-thickness expansion function. Figure 6b and Table 3 show the outcomes of the different ESL models and their comparison with the LW Coarse discretization. The ESL approach provides a closer $\varepsilon _{xx}$ to that obtained with ABQ. Additionally, all ESL models except ESL-TE 1 exhibit a similar $\varepsilon _{xx}$ distribution along the *y*-axis, particularly in the region near $y=a/2$ where $\varepsilon _{xx}$ decreases; see Fig. 6b. ESL-TE 1 perfectly matches the $\varepsilon _{xx}$ distribution of the ABQ model, especially near the edge, where the other models show edge effects. The stress distribution prediction has not been reported in this document as it has already been shown in previous works by the authors; see [34, 49].

**Table 2:** Mesh convergence on the $\varepsilon _{xx}$ at the plate hole. Each CUF model employs a 1LE3 expansion per layer

Model	DOF	$\varepsilon _{xx}\cdot 10^2$
ABQ2D Coarse	12408	3.47
ABQ2D Refined	28044	3.41
LW Coarse	86229	3.47
LW Refined	173121	3.48

**Table 3:** Effect of the structural theory on the $\varepsilon _{xx}$ at the plate hole. Each model employs the coarse mesh

Model	DOF	$\varepsilon _{xx}\cdot 10^2$
ESL-TE 1	13266	3.36
ESL-TE 2	19899	3.42
ESL-TE 3	26532	3.40
ESL-TE 4	33165	3.40
LW Coarse	86229	3.47

### Concentration Factor Minimization

This section shows results concerning the minimization of the Concentration Factor (CF) around the cutout, that is

15 $$\begin{aligned} CF1=\frac{\varepsilon _{xx|\text {cutout}}}{\varepsilon _{xx|\text {edge}}}, \hspace{5.0pt}\hspace{5.0pt}CF2=\sigma _{xx|\text {cutout}}. \end{aligned}$$

The first expression involves the ratio of the strains evaluated at the cutout and the edge, whereas the second only considers the $\sigma _{xx}$ component evaluated at the cutout. It is therefore to expect differences in the optimization results as two different objective functions are considered.

The optimization problem is formulated as follows:

16 $$\begin{aligned} \underset{{\textbf{x}}}{\text {min}} \hspace{5.0pt}CF({\textbf{x}}), \end{aligned}$$

in which ${\textbf{x}}=\{T_0^1,T_1^1,T_0^2,T_1^2\}$ represents the design variables vector, and the stacking sequence is expressed as $\theta =[\langle T_0^1,T_1^1\rangle ,\langle T_0^2,T_1^2\rangle ]_s$. Each design variable’s lower and upper bound in ${\textbf{x}}$ are $-90^\circ$ and $90^\circ$, respectively. Note that the selected design variables can vary continuously within the prescribed range. These bounds apply to the remaining optimization cases in the manuscript. The optimization problem is solved through modeFrontier$^{\textcircled {c}}$ [50], a multidisciplinary software platform for process automation and optimization. In particular, the chosen optimization algorithm is a metaheuristic one. Since, to the best of the authors’ knowledge, no proof of convergence is available in the literature for metaheuristic algorithms, the found solutions should be referred to as near- or pseudo-optimal. Despite that, for conciseness, the found solutions are referred to as optimal throughout the manuscript. The utilized algorithm combines global and local search capabilities to enhance the efficiency and robustness of the optimization process. Initially, it conducts a global search to explore the design space and identify promising regions. This process involves leveraging previously evaluated designs to guide the search efficiently. By performing virtual optimizations using a response surface constructed from the previous designs, the algorithm can quickly identify candidate solutions, which are validated through detailed evaluations, i.e., actual FE simulations. This iterative approach, where newly evaluated designs continually improve the knowledge base, allows the algorithm to adapt and refine its search strategy over time, ultimately leading to increasingly accurate and optimal solutions. Note that the original population and random seed utilized for the global search phase are the same for all the structural models considered.

**Fig. 7:**
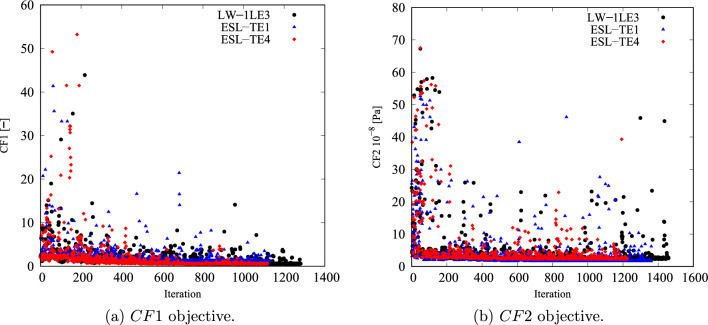
Objective function versus iteration for the different cost functions and structural theories for the *CF* unconstrained optimization problem

Figure 7 shows the cost function convergence. The first hundred to two hundred iterations are to the global search; the local search starts and reaches convergence afterward. Nonetheless, isolated cost function evaluations are visible outside of the convergence region and are new designs proposed by the algorithm that is evaluated to avoid entrapment in local minima and increase the accuracy of the underlying response surface. In the *CF*1 case, convergence is reached after 700–800 iterations, whereas *CF*2 converges after 800 iterations.

**Fig. 8:**
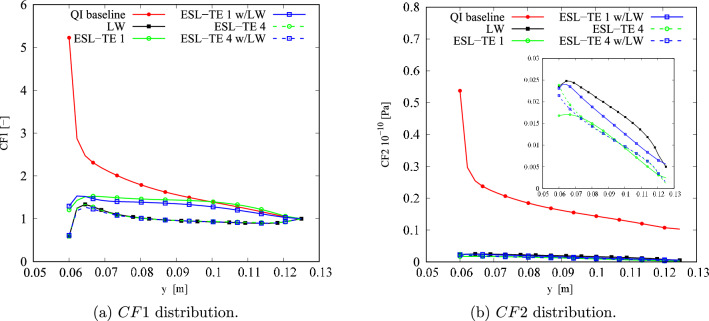
Optimal *CF* distributions for the different objective functions and structural theories for the unconstrained problem

**Fig. 9:**
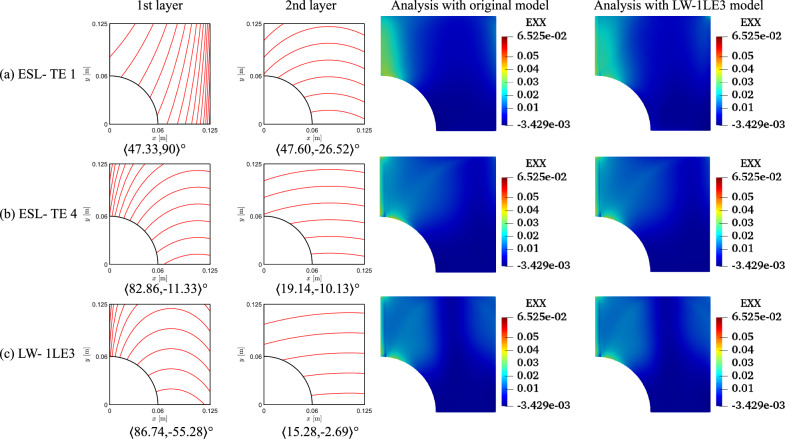
Fiber paths and $\varepsilon _{xx}$ contours of the *CF*1 unconstrained optimization

**Fig. 10:**
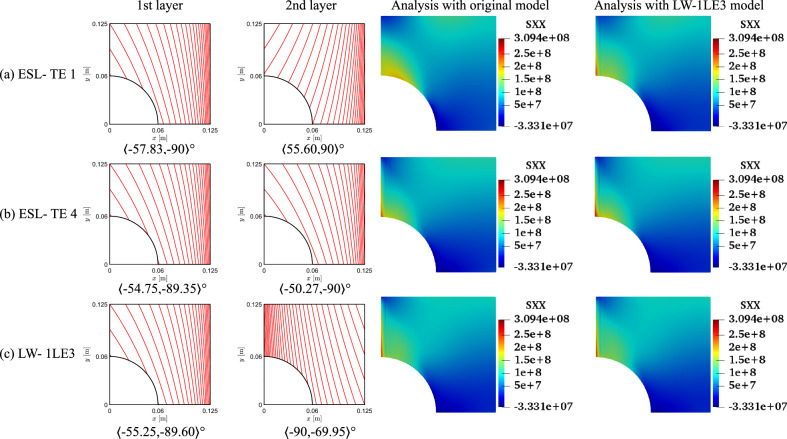
Fiber paths and $\sigma _{xx}$ contours of the *CF*2 unconstrained optimization

The results of the unconstrained problems for the different structural models are gathered in Table 4. The outcomes are compared against a quasi-isotropic (QI) plate with $[90,\pm 45,0]_s$ layup and the same dimensions, boundary, and loading conditions of the VAT plate under investigation. The QI presents *CF*1 and *CF*2 values equal to 5.23 and 53.76$\cdot 10^8$ Pa, respectively. The obtained optimal results present a significant reduction of both *CF* definitions. The *CF*1 case presents a 76–89% decrease, while a 96% reduction is observed for *CF*2. The *CF* trends are available in Fig. 8. Regarding the *CF*1 index, the ESL-TE 1 design presents an initial rise, reaching a plateau and dropping toward the edge of the plate. The ESL-TE 4 and LW optimal present an identical trend, rising close to the cutout and plateauing about *CF*1 equal to 1. The retrieved optimal designs for *CF*2 present distributions one order of magnitude lower than the QI design and are available in the zoomed region in Fig. 8b. Conversely to the *CF*1 case, the ESL-TE 1 optimum presents a different distribution when this design is evaluated with an LW-1LD3 structural theory. Such discrepancies are not so relevant in the ESL-TE 4 optimum, where a slight difference in the peak value at the cutout is evidenced. Oppositely to *CF*1, the ESL-TE 4 and LW do not match because of the different modeling approaches. Figures 9 and 10 show the optimized fiber paths and the $\varepsilon _{xx}$ and $\sigma _{xx}$ contours. A concentration is nearby to the $y=0$ symmetry plane for the ESL-TE 1 optima. These regions are wider when such designs are evaluated with the lower-order ESL model. The ESL-TE 4 and LW optimal present similar contours. Overall, the three structural models tend to redistribute the *CF* toward the upper right corner, which is more evident in the $\sigma _{xx}$ contour, as shown in the figures.

**Table 4:** Optimal designs for the unconstrained *CF* optimization problem. The reported *CF*1 is dimensionless, whereas the *CF*2 case is expressed in Pa. The superscripts indicate the relative difference between the current design and the QI baseline

	*CF*1			*CF*2		
	ESL-TE 1	ESL-TE 4	LW-1LE3	ESL-TE 1	ESL-TE 4	LW-1LE3
$\langle T_0^1,$	$\langle 47.33,$	$\langle 82.86,$	$\langle 86.74,$	$\langle -57.83,$	$\langle -54.75,$	$\langle -55.25,$
$T_1^1 \rangle$ $[^\circ ]$	$90\rangle$	$-11.33\rangle$	$-55.28\rangle$	$-90\rangle$	$-89.35\rangle$	$-89.60\rangle$
$\langle T_0^2,$	$\langle 47.60,$	$\langle 19.14,$	$\langle 15.28$	$\langle 55.60,$	$\langle -50.27,$	$\langle -90$
$T_1^2\rangle$ $[^\circ ]$	$-26.52\rangle$	$-10.13\rangle$	$-2.69\rangle$	$90\rangle$	$-90\rangle$	$-69.95\rangle$
*CF* [-/$\cdot 10^{-8}$ Pa]	$1.205^{-76.96\%}$	$0.592^{-88.68\%}$	$0.586^{-88.79\%}$	$1.678^{-96.87\%}$	$2.384^{-95.56\%}$	$2.356^{-95.61\%}$
*CF* evaluated with an LW-1LE3 model						
*CF* [-/$\cdot 10^{-8}$ Pa]	$1.296^{-75.22\%}$	$0.612^{-88.29\%}$	$0.586^{-88.79\%}$	$2.307^{-95.70\%}$	$2.385^{-95.56\%}$	$2.356^{-95.61\%}$

Despite the significant reduction of both *CF*, the optimal designs cannot be manufactured as they exceed the maximum curvature allowed. Therefore, the constrained optimization is posed as follows:

17 $$\begin{aligned} \underset{{\textbf{x}}}{\text {min}} \hspace{5.0pt}CF({\textbf{x}}) \hspace{5.0pt}\text {s.t.} \hspace{5.0pt}\hspace{5.0pt}-1/r_{\text {min}}\le \kappa ^k({\textbf{x}}) \le 1/r_{\text {min}} \hspace{5.0pt}k=1,2. \end{aligned}$$

**Fig. 11:**
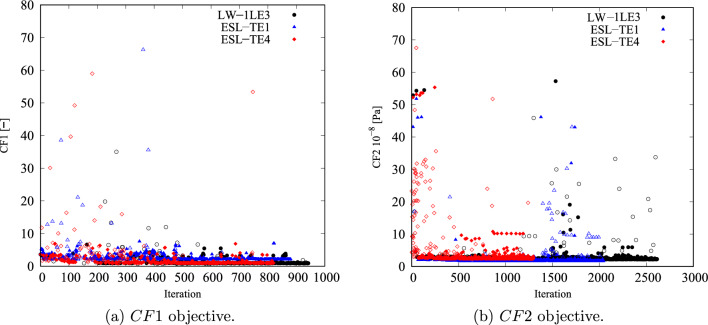
Objective function versus iteration for the different cost functions and structural theories for the *CF* constrained optimization problem. Filled and empty markers correspond to feasible and unfeasible designs, respectively

**Fig. 12:**
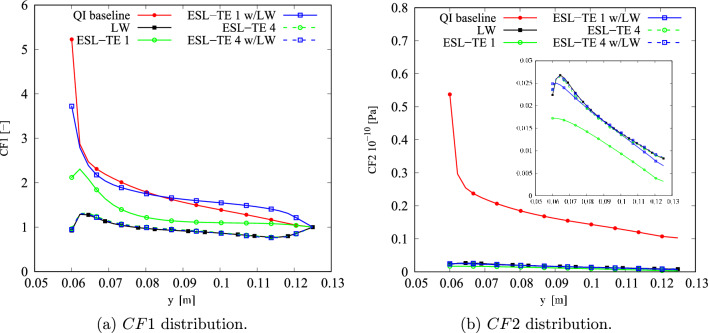
Optimal *CF* distributions for the different objective functions and structural theories for the constrained problem

**Fig. 13:**
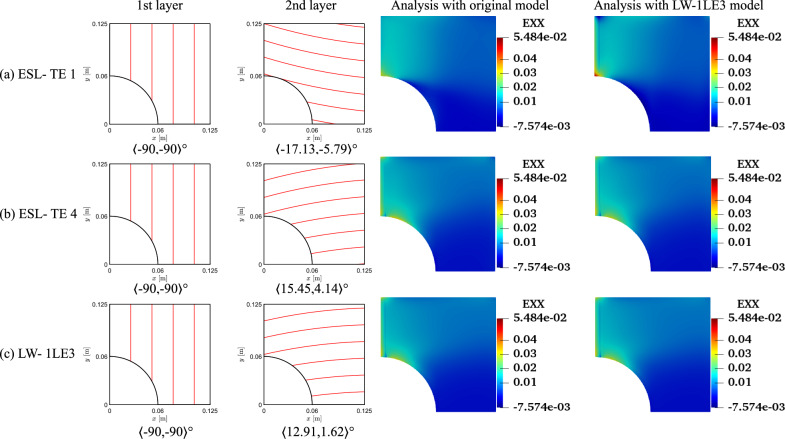
Fiber paths and $\varepsilon _{xx}$ contours of the *CF*1 constrained optimization

**Fig. 14:**
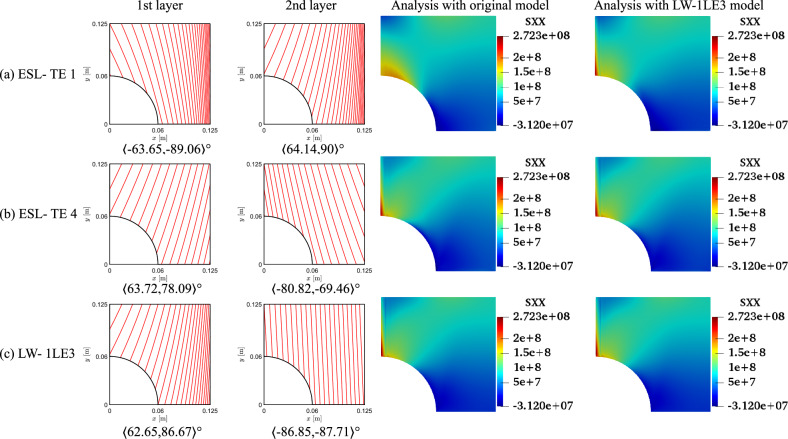
Fiber paths and $\sigma _{xx}$ contours of the *CF*2 constrained optimization

Figure 11 presents the convergence of the two *CF* cases. It is worth mentioning that the cost function evaluations from the unconstrained problems were filtered based on the curvature value. The retrieved solutions to this problem are listed in Table 5. The constrained designs significantly reduce both *CF* objective functions, with the first case ranging from 59 to 82% decrease and *CF*2 presenting around a 96% reduction. Figure 12 shows the *CF* distributions along the *y*-direction. The *CF*1 ESL-TE 1 optimum presents a sudden increase close to the hole and subsequent drop, reaching a stationary value close to 1. However, when this optimum is evaluated with LW kinematics, the *CF*1 is a monotonically decreasing function. Then, as in the unconstrained optimization, the ESL-TE 4 and LW optima present identical trends as their retrieved optimal stacking sequences are very similar. The zoomed area in Fig. 12b shows the *CF*2 trends of the optimal solutions. Differences are appreciated when the ESL-TE 1 design is evaluated with lower-order ESL and LW kinematics. Instead, the ESL-TE 4 optimum does not present such discrepancies and matches the *CF*2 trend of the LW solution. Figures 13 and 14 present the optimized fiber paths and the $\varepsilon _{xx}$ and $\sigma _{xx}$ contours. As in the unconstrained problem, ESL-TE 1 presents a broad concentration region close to the cutout, making such a region broader when evaluating the optimal design with the lower-order structural model. Conversely, the contours produced with the optimal ESL-TE 4 and LW solutions are almost identical. Again, a redistribution of the *CF* toward the upper right edge is observed, especially the *CF*2 index.

**Table 5:** Optimal designs for the constrained *CF* optimization problem. The reported *CF*1 is dimensionless, whereas the *CF*2 case is expressed in Pa. The superscripts indicate the relative difference between the current design and the QI baseline

	*CF*1			*CF*2		
	ESL-TE 1	ESL-TE 4	LW-1LE3	ESL-TE 1	ESL-TE 4	LW-1LE3
$\langle T_0^1,$	$\langle -90,$	$\langle -90,$	$\langle -90,$	$\langle -63.65,$	$\langle 63.72,$	$\langle 62.65,$
$T_1^1 \rangle$ $[^\circ ]$	$-90\rangle$	$-90\rangle$	$-90\rangle$	$-89.06\rangle$	$78.09\rangle$	$86.67\rangle$
$\langle T_0^2,$	$\langle -17.13,$	$\langle 15.45,$	$\langle 12.91,$	$\langle 64.14,$	$\langle -80.82,$	$\langle -86.85,$
$T_1^2\rangle$ $[^\circ ]$	$-5.79\rangle$	$4.14\rangle$	$1.62\rangle$	$90\rangle$	$-69.46\rangle$	$-87.71\rangle$
*CF* [-/$\cdot 10^{-8}$ Pa]	$2.110^{-59.65\%}$	$0.964^{-81.56\%}$	$0.923^{-82.35\%}$	$1.723^{-96.79\%}$	$2.354^{-95.62\%}$	$2.241^{-95.83\%}$
$\kappa _1/\kappa _2$ [$\text {m}^{-1}$]	0.00/1.5748	0.00/1.5748	0.00/1.5748	1.5748/1.5748	0.888/0.558	1.540/0.006
*CF* evaluated with an LW-1LE3 model						
*CF* [-/$\cdot 10^{-8}$ Pa]	$3.723^{-28.81\%}$	$0.946^{-81.91\%}$	$0.923^{-82.35\%}$	$2.489^{-95.37\%}$	$2.359^{-95.61\%}$	$2.241^{-95.83\%}$

The fiber paths illustrated in Figs. 9 and 10 present discrepancies, as expected, because of the different objective functions considered. Furthermore, the various structural theories for each *CF* function lead to distinct patterns. Such discrepancies among optimal solutions for *CF*1 and *CF*2 and structural models are also present in the constrained cases; see Figs. 13 and 14. The main reason for such differences stems from the structural theory employed to compute the objective function and where these quantities are evaluated. Recalling Eq. (15), the strains and stresses involved are calculated at the cutout and the upper edge of the perforated plate. These constitute free-edge regions where it is well known that classical models such as the ESL-TE 1, essentially an Abaqus FSDT-like model, fail at predicting strains and stresses. Then, as higher-order models are utilized, those predictions become more accurate. Indeed, it is observed that the ESL-TE 4 and LW models provide similar fiber paths, although differences in the design variables are appreciated. Such diverse optimal stacking sequences are especially observed when *CF*2 is considered. Recalling the generalized Hooke’s law, the $\sigma _{xx}$ term involves the out-of-plane strain components, whose prediction is more accurate as the kinematic model is more refined.

Regarding the constrained solutions, it is worth noting that *CF*1 designs present straight fibers oriented to $-90^\circ$ in the outer layers, while internal plies show active constraint values, i.e., $\kappa _2=\kappa _{max}=1.5748$ $\text {m}^{-1}$. On the other hand, *CF*2 optimal presents only active constraints in the ESL-TE 1 design, while higher-order models do not. In addition, the LW $\langle T_0^2,T_1^2\rangle$ lamination is barely steered. Finally, it is remarkable that in *CF*2, the $\langle T_0^1,T_1^1\rangle$ of the different structural models present similar fiber paths.

### Strength Maximization

**Fig. 15:**
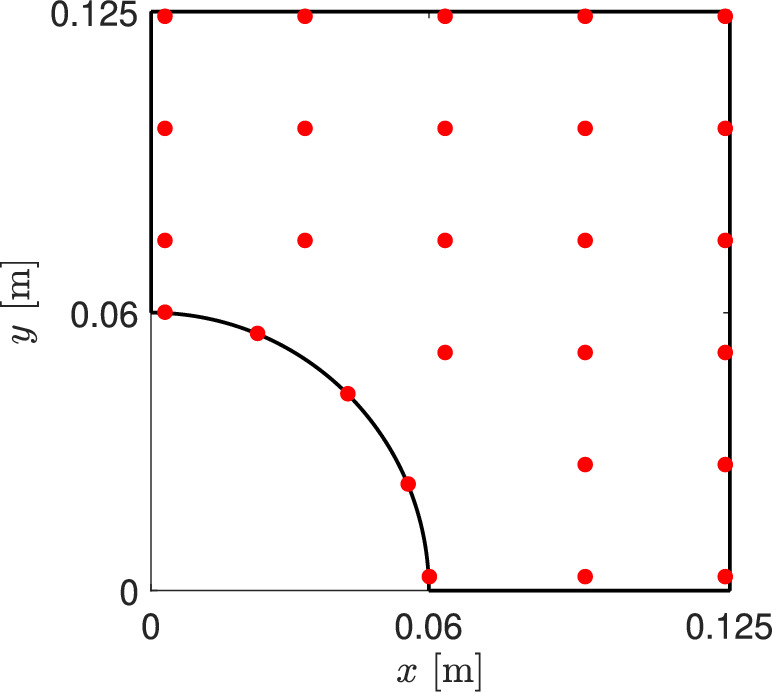
Control points in which the $FI$ are evaluated trough the thickness to perform the strength maximization

The cutout plate under stretching, whose *CF* was optimized in the previous section, is now considered to conduct a maximization of the safety factor *SF*. As stated in the work by Groenwold and Haftka [27], minimizing the maximum failure index *FI* is equivalent to maximizing the minimum safety factor. This problem can be posed as follows:

18 $$\begin{aligned} \underset{{\textbf{x}}}{\text {max}} \hspace{5.0pt}\text {min}(SF_j({\textbf{x}})) \hspace{5.0pt}j=1,\ldots ,n_s, \end{aligned}$$

where $n_s$ represents the number of sampling points through the thickness where *SF* is measured. In this work, the Hashin 3D failure criteria [51] is evaluated through the thickness at the control points highlighted in Fig. 15, and the *SF* is calculated according to the procedure depicted in the Appendix A. Points were selected to cover most of the plate area without requiring excessive postprocessing operations, which would ultimately slow down the optimization process. For each point, twelve values were evaluated in the thickness direction.

Hashin 3D establishes four failure modes, namely, Fiber Tension (FT), Fiber Compression (FC), Matrix Tension (MT), and Matrix Compression (MC). The material strengths, taken from Toray$^{\textcircled {c}}$ T800S datasheet [52], are listed in Table 6.

**Table 6:** Strength properties of the material used for strength optimization. Taken from Toray$^{\textcircled {c}}$ T800S datasheet [52]

$X_{T}$ [MPa]	3290.0
$X_C$ [MPa]	1490.0
$Y_T$ [MPa]	79.0
$Y_C$ [MPa]	300.0
$S_{12}$ [MPa]	135.0
$S_{13}=S_{23}$ [MPa]	87.6

**Fig. 16:**
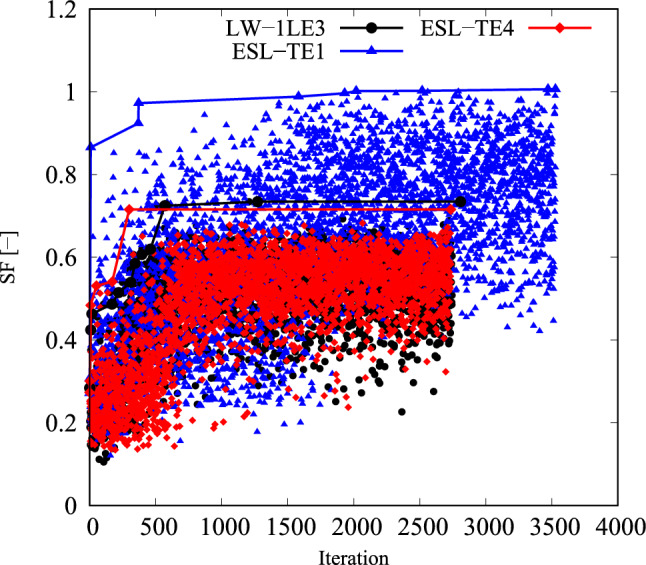
*SF* objective function versus iteration for the different structural theories in the unconstrained problem. Markers indicate all evaluated designs, while solid lines with markers show the evolution of the best design as iterations progress

As stated before, the cost function in Eq. (18) is non-convex and is based on four failure modes. Because of that, a classic GA available in modeFrontier$^{\textcircled {c}}$ is chosen as the optimization algorithm. Instead of using the algorithm that combines global and local search capabilities, the GA is preferred in this case, because the location, in terms of the in-plane coordinates *x* and *y*, of the minimum *SF* and the failure index providing that it may vary between individual iterations. The initial population and the random seed were kept constant for the three structural theories involved. Additionally, the autonomous mode was selected. This means that the algorithm uses the information gathered from the problem analysis to drive the optimization in the right direction and stops when the optimal design does not improve any further. The convergence of the optimization process is shown in Fig. 16. The solid lines follow the improvement of the best design found, while individual markers correspond to the evaluated population. No further improvement is found from the thousandth iteration onwards for ESL-TE 4 and LW, while ESL-TE 1 requires two thousand more evaluations to find its best solution.

**Fig. 17:**
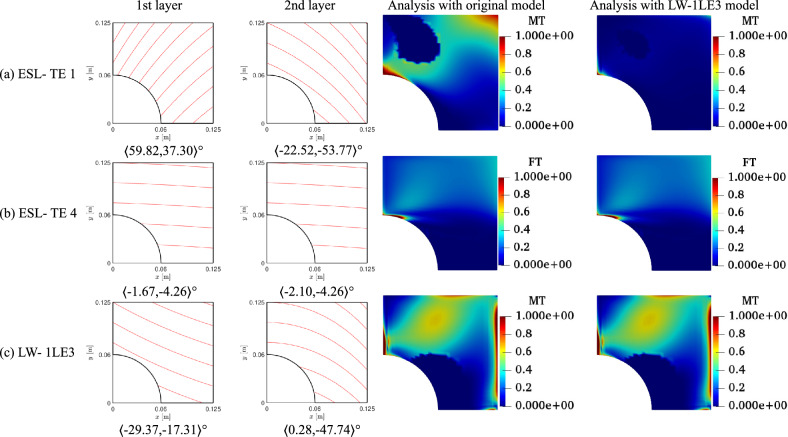
Fiber patterns and *FI* contours of solutions for unconstrained optimization. ESL design contours computed with original ESL and LW-1LE3 models, showing the critical failure mode

As in Sect. 4.2, ESL-TE 1, ESL-TE 4, and LW-1LE3 models are considered to solve the optimization problem. Table 7 gathers the optimal solutions to the unconstrained optimization problem. Figure 17 shows the fiber paths and the *FI* contours. From Table 7, one can observe that the different theories lead to a diversity of optimal designs. Additionally, the safety factors vary for each model. While the *SF* of the ESL-TE 1 decreases by 66%, that of the ESL-TE 4 presents practically the same value. It is remarkable that when the optimum ESL designs are evaluated with an LW-1LE3 model, the value of *SF* diminishes, being more evident for the ESL-TE 1 design. The point where the failure mechanism is triggered is retrieved by following the procedure depicted in the Appendix A for the $n_s$ sampling points, and the *FI* of the failure mechanics is in Fig. 17. The ESL-TE 1 model is affected by MT close to the cutout and the top of the laminate. However, when this design is evaluated with an LW-1LE3 structural theory, failure occurs at the cutout (on the symmetry plane) and the bottom of the stack. Concerning ESL-TE 4, failure onset is due to FT at the cutout and $z=h/2$. Nonetheless, when an LW-1LE3 model is employed for this design, the FT occurs at the cutout and $z=h/2$. The LW-1LE3 optimal design fails due to MT at the edge where the load is applied, at the interface between the first and second layers.

**Table 7:** Optimal designs for the unconstrained strength optimization problem

	ESL-TE 1	ESL-TE 4	LW-1LE3
$\langle T_0^1,$	$\langle 59.82,$	$\langle -1.67,$	$\langle -29.37,$
$T_1^1 \rangle$ $[^\circ ]$	$37.30\rangle$	$-4.26\rangle$	$-17.31\rangle$
$\langle T_0^2,$	$\langle -22.52,$	$\langle -2.10,$	$\langle 0.28,$
$T_1^2\rangle$ $[^\circ ]$	$-53.77\rangle$	$-4.26\rangle$	$-47.74\rangle$
*SF* [-]	1.006(FT)	0.715(FT)	0.734(MT)
*SF* evaluated with an LW-1LE3 model			
*SF* [-]	0.340(MT)	0.713(FT)	0.734(MT)

Due to the turning radius limitations, the constrained optimization problem has to be considered. The constrained safety factor maximization problem reads as follows:

19 $$\begin{aligned} \underset{{\textbf{x}}}{\text {max}} \hspace{5.0pt}\text {min}(SF_j({\textbf{x}})) \hspace{5.0pt}j=1,\ldots ,n_s, \hspace{5.0pt}\text {s.t.} \hspace{5.0pt}\hspace{5.0pt}-1/r_{\text {min}}\le \kappa ^k({\textbf{x}}) \le 1/r_{\text {min}} \hspace{5.0pt}k=1,2. \end{aligned}$$

**Fig. 18:**
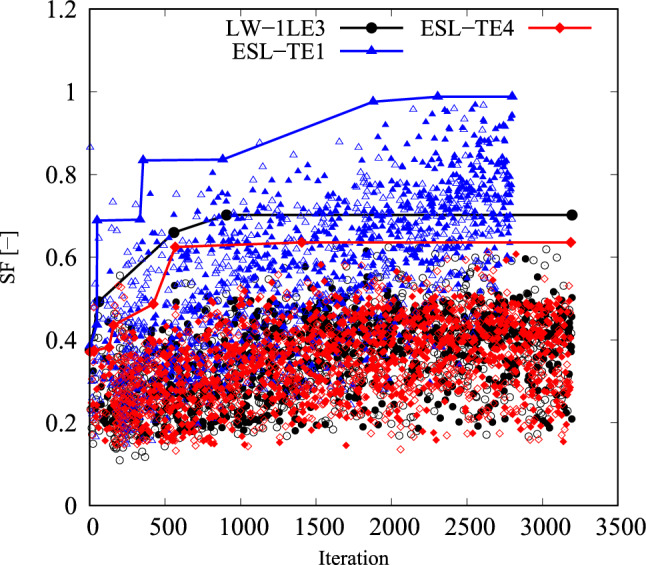
*SF* objective function versus iteration for the different structural theories in the constrained problem. Markers indicate all evaluated designs, while solid lines with markers show the evolution of the best design as iterations progress. Filled and empty markers correspond to feasible and unfeasible designs, respectively

The convergence of the solutions to the previous problem are available in Fig. 18. The designs fulfilling the curvature constraint are represented with solid markers, while the empty ones correspond to unfeasible designs. The ESL-TE 4 and LW structural models found their best solution between a thousand and a thousand and five hundred evaluations, while the ESL-TE 1 required additional generations.

**Fig. 19:**
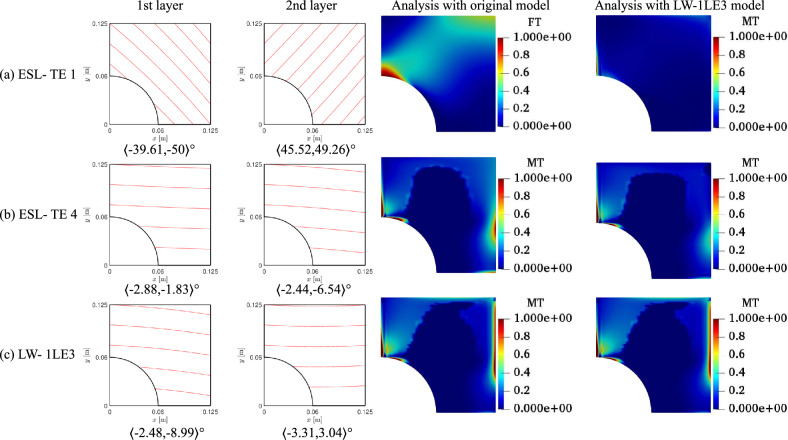
Fiber patterns and $FI$ contours of solutions for constrained optimization. ESL design contours computed with original ESL and LW-1LE3 models, showing the critical failure mode

The optimal solutions to the constrained problem are listed in Table 8. Figure 19 displays the fiber paths and the *FI* contours. It is evidenced again that different structural theories lead to nonidentical optima. The optimal ESL-TE 1 changes the failure mechanism from MT in the unconstrained problem to FT in the constrained case. The failure onset is reached at the cutout and bottom of the stack. However, when this design is evaluated with an LW-1LE3 model, the *SF* equals 0.384, presenting a 61% difference compared to the initially predicted *SF*. Besides, the onset occurs at the midplane of the cutout. The ESL-TE 4 solution fails due to MT at the right edge, in the third layer of the stack. Nonetheless, if the LW-1LE3 is used, failure will occur in a similar position on the right edge but in the laminate’s midplane, and the *SF* is slightly higher than the *SF* computed by the ESL-TE 4 model. The LW-1LE3 constrained design fails due to MT at the right edge and the second layer.

The ESL-TE 1 fiber paths are illustrated in Figs. 17 and 19 present similarities with those obtained for the strength optimization in the work by Ding et al. [53] despite the discrepancies in steering variation laws, geometry, and loading conditions. The authors employed an FSDT model and the strain-based Tsai–Wu failure criterion in that work. The strength designs by [53] present fiber paths sourcing from the interior corner and pointing toward the outer corner when an in-plane load is applied. Such a pattern is also present in the current ESL-TE1 designs. However, differences between the lower-order ESL and the higher-order ESL and LW in the present document are evident, as observed in Figs. 17 and 19. This contrast is due to the nature of the optimization problem, where stresses are involved in the objective function. Indeed, the various structural theories share the same initial sampling of the optimization processes. It is, therefore, the effect of the structural theory in the prediction of the stress state and hence the *SF* which influences the found optimal design.

Finally, it is worth remarking that the constrained solutions present non-active constraint values, i.e., the requirements on $\kappa _1$ and $\kappa _2$ are not equal to the maximum permitted value of $\kappa _{max} = 1.5748$ $\text {m}^{-1}$. From a mathematical perspective, this means that the maximum turning radius might not be necessary when optimizing the strength of the VAT laminate. This assumption applies only in the present case, where the manufacturing signature of the AFP process is not considered within the numerical model, and where the dimensions of the plate are very small.

**Table 8:** Optimal designs for the constrained strength optimization problem

	ESL-TE 1	ESL-TE 4	LW-1LE3
$\langle T_0^1,$	$\langle -39.61,$	$\langle -2.88,$	$\langle -2.48,$
$T_1^1 \rangle$ $[^\circ ]$	$-50\rangle$	$-1.83\rangle$	$-8.99\rangle$
$\langle T_0^2,$	$\langle 45.52,$	$\langle -2.44,$	$\langle -3.31,$
$T_1^2\rangle$ $[^\circ ]$	$49.26\rangle$	$-6.54\rangle$	$3.04\rangle$
*SF* [-]	0.987(FT)	0.636(MT)	0.702(MT)
$\kappa _1/\kappa _2$ [$\text {m}^{-1}$]	1.11/0.36	0.14/0.57	0.91/0.89
*SF* evaluated with an LW-1LE3 model			
*SF* [-]	0.384(MT)	0.649(MT)	0.702(MT)

## Conclusion

This document investigated the concentration factor and strength optimization problems involving perforated VAT composite plates, with and without including manufacturing limitations. In detail, the concentration factor optimization was studied by considering two different metrics: (i) a ratio between strains measured at the cutout and the free edge (*CF1* case), and (ii) the stress component in the pulling direction evaluated at the hole (*CF2* case). The strength maximization was conducted utilizing the safety factor calculated using the Hashin 3D failure criteria. The plate was analyzed using ESL and LW CUF-based models. The optimization problems were solved through modeFrontier$^{\textcircled {c}}$. Specifically, the concentration factor problems utilized an algorithm that combines global and local search capabilities, while the strength maximization employed a genetic algorithm. The problems faced in this manuscript are non-convex and may have multiple local minima. The starting generations and random seeds were kept constant for each optimization problem and structural theory to investigate the role of the structural theory. The study identified optimal designs for each structural theory and optimization problem.

According to the outcomes gathered in this paper, the following comments can be made:

1. The two metrics employed for the concentration factor minimization led to different optimal fiber paths, as expected. Both indices outperformed the concentration factors of the QI plate considered as the baseline design. The *CF1* case presented a reduction between 60 and 89%, while the *CF2* provided around a 95% decrease. Thus, a stress-based objective function is preferable when minimizing the strain.
2. Both* CF1* and *CF2* optimizations retrieved different stacking sequences for the various structural theories involved, although there were some similarities in the fiber path trend for individual layers; see Tables 4 and 5. These discrepancies are more evident between lower-order ESL models, such as the FSDT-like TE 1, and higher-order kinematic models. Those differences were less pronounced between the ESL-TE 4 and LW models employed.
3. When maximizing strength, the different models led to diverse fiber paths in the unconstrained and constrained problems. Additionally, some structural models presented nearly unsteered solutions. This suggests that the constraint on maximum steering curvature may not be necessary for this optimization problem, especially when the plate dimensions are small.
4. In general, when optimizing the concentration factor and strength of perforated and relatively thick plates, one should employ higher-order ESL and LW models, since it is noted that lower-order structural theories do not capture well either free-edge strains and stresses or out-of-plane stress components used for the computation of 3D failure indices.
5. A possible solution to mitigate the computational burden associated with using higher-order ESL and LW models for concentration factor and strength optimization is the development of global–local, node-dependent kinematics, or multi-fidelity machine learning strategies in regions where measuring these magnitudes is of interest.

Future research will not only consider the curvature manufacturing constraint; it will also incorporate the actual modeling of manufacturing defects, such as gaps and overlaps, as these play an important role in the mechanical performance of the tow-steered laminate. Despite their significance, modeling these defects was not the scope of the present document. This paper considers relatively simple composite systems, e.g., a four-ply symmetric stack with four design variables, that may not represent a realistic structure. Such a choice does not affect the scope of this work, which is to address the influence of the structural theory on the optimal solution. In future works, larger problems with tens to thousands of design variables and more complex fiber paths may be considered. In that case, gradient-based optimization algorithms could speed up the tailoring process. Another strategy to consider to improve efficiency is adopting of surrogate models. Also, the least-weight optimization of steered laminates, including defect modeling and mechanical constraints, will be investigated. In-situ effects will be considered to exploit CUF ability to obtain very accurate local strain and stress fields.

## Data Availability

The data of this paper are available on request.

## Appendix A: Failure index and strength constraint formulation

The Hashin 3D failure criteria [51] predict the onset within a laminated structure. Moreover, it provides information regarding the failure mechanism that is triggered. These are expressed in the following:

1. Fiber tension:
  20 $$\begin{aligned} FI:= \left( \frac{\sigma _{11}}{X_T}\right) ^2+\frac{\sigma _{12}^2+\sigma _{13}^2}{S_{12}^2}. \end{aligned}$$
2. Fiber compression
  21 $$\begin{aligned} FI:= \left( \frac{\sigma _{11}}{X_C}\right) ^2. \end{aligned}$$
3. Matrix tension:
  22 $$\begin{aligned} FI:= \frac{(\sigma _{22}+\sigma _{33})^2}{Y_T^2}+\frac{\sigma _{23}^2-\sigma _{22}\sigma _{33}}{S_{23}^2}+\frac{\sigma _{12}^2+\sigma _{13}^2}{S_{12}^2}. \end{aligned}$$
4. Matrix compression:
  23 $$\begin{aligned} & FI:= \left[ \left( \frac{Y_C}{2S_{23}}\right) ^2-1\right] \left( \frac{\sigma _{22}+\sigma _{33}}{Y_C}\right) +\frac{(\sigma _{22} +\sigma _{33})^2}{4S_{23}^2}\nonumber \\ & \quad +\frac{\sigma _{23}^2-\sigma _{22}\sigma _{33}}{S_{23}^2}+\frac{\sigma _{12}^2+\sigma _{13}^2}{S_{12}^2}, \end{aligned}$$

where $\sigma _{ij}$ are the stress tensor components in the material coordinate system. Direction 1 corresponds to the fiber direction, while 2 and 3 represent the transverse directions. In addition, *X* and *Y* are the material strengths; subscripts *T* and *C* denote tension and compression, respectively, and $S_{ij}$ are the material shear strengths. Since linear analyses are considered, these components can be expressed in terms of a safety factor *SF*, which is equal to the scalar load multiplier $\lambda$ that results in the failure onset. The stress tensor at which any of the above $FI=1$ can be expressed as

$$\begin{aligned} \boldsymbol{{\sigma }}=\lambda {\tilde{\boldsymbol{\sigma }}}. \end{aligned}$$

Equations (20) to (23) can be rewritten at the failure onset as

1. Fiber tension:
  24 $$\begin{aligned} FI=\lambda ^2\left[ \left( \frac{{\tilde{\sigma }}_{11}}{X_T}\right) ^2+\frac{{\tilde{\sigma }}_{12}^2+{\tilde{\sigma }}_{13}^2}{S_{12}^2}\right] =a\lambda ^2=1. \end{aligned}$$
2. Fiber compression:
  25 $$\begin{aligned} FI=\lambda ^2\left( \frac{{\tilde{\sigma }}_{11}}{X_C}\right) ^2=a\lambda ^2=1. \end{aligned}$$
3. Matrix tension:
  26 $$\begin{aligned} FI=\lambda ^2\left[ \frac{({\tilde{\sigma }}_{22}+{\tilde{\sigma }}_{33})^2}{Y_T^2}+\frac{{\tilde{\sigma }}_{23}^2-{\tilde{\sigma }}_{22}{\tilde{\sigma }}_{33}}{S_{23}^2}+\frac{{\tilde{\sigma }}_{12}^2+{\tilde{\sigma }}_{13}^2}{S_{12}^2}\right] =a\lambda ^2=1. \end{aligned}$$
4. Matrix compression:
  27 $$\begin{aligned} \begin{aligned} FI=&\lambda ^2\left[ \frac{({\tilde{\sigma }}_{22}+{\tilde{\sigma }}_{33})^2}{4S_{23}^2}+\frac{{\tilde{\sigma }}_{23}^2-{\tilde{\sigma }}_{22}{\tilde{\sigma }}_{33}}{S_{23}^2}+\frac{{\tilde{\sigma }}_{12}^2+{\tilde{\sigma }}_{13}^2}{S_{12}^2} \right] + \\&+\lambda \left[ \left( \frac{Y_C}{2S_{23}}\right) ^2-1\right] \left( \frac{\sigma _{22}+\sigma _{33}}{Y_C}\right) =a\lambda ^2+b\lambda =1. \end{aligned} \end{aligned}$$

These quadratic equations can be easily solved, and the safety factor *SF* corresponds with the smallest positive root of $\lambda$.

## Abbreviations

ABQ: Abaqus$^{\textcircled {c}}$ software
CF: Concentration Factor
CFF: Continuous Filament Fabrication
CTS: Continuous Tow Shearing
CUF: Carrera Unified Formulation
CLPT: Classical Laminated Plate Theory
ESL: Equivalent-Single-Layer
FE: Finite Element
FC: Fiber Compression
FN: Fundamental Nucleus
FT: Fiber Tension
FFF: Fused Filament Fabrication
FSDT: First-order Shear Deformation Theory
GA: Genetic Algorithm
LW: Layer-Wise
LE: Lagrange Expansion
MC: Matrix Compression
MT: Matrix Tension
PSO: Particle Swarm Optimization
PVD: Principle of Virtual Displacements
QI: Quasi-isotropic
TE: Taylor Expansion
VAT: Varible Angel Tow
VSC: Variable Stiffness Composites
